# Early corticosteroid dose tapering in patients with acute exacerbation of idiopathic pulmonary fibrosis

**DOI:** 10.1186/s12931-022-02195-3

**Published:** 2022-10-26

**Authors:** Keisuke Anan, Yuki Kataoka, Kazuya Ichikado, Kodai Kawamura, Takeshi Johkoh, Kiminori Fujimoto, Kazunori Tobino, Ryo Tachikawa, Hiroyuki Ito, Takahito Nakamura, Tomoo Kishaba, Minoru Inomata, Tsukasa Kamitani, Hajime Yamazaki, Yusuke Ogawa, Yosuke Yamamoto

**Affiliations:** 1 Department of Healthcare Epidemiology, Graduate School of Medicine, Kyoto University, Yoshida Konoe-cho Sakyo-ku, 606-8501 Kyoto-City, Japan; 2 Division of Respiratory Medicine, Saiseikai Kumamoto Hospital, Kumamoto, Japan; 3 Systematic Review Workshop Peer Support Group (SRWS-PSG), Osaka, Japan; 4 Department of Internal Medicine, Kyoto Min-Iren Asukai Hospital, Kyoto, Japan; 5 Section of Clinical Epidemiology, Department of Community Medicine, Graduate School of Medicine, Kyoto University, Kyoto, Japan; 6 Department of Radiology, Kansai Rosai Hospital, Hyogo, Japan; 7 Department of Radiology, Kurume University School of Medicine, Fukuoka, Japan; 8 Department of Respiratory Medicine, Iizuka Hospital, Fukuoka, Japan; 9 Department of Respiratory Medicine, Kobe City Medical Center General Hospital, Hyogo, Japan; 10 Department of Pulmonology, Kameda Medical Center, Chiba, Japan; 11 Department of General Internal Medicine, Nara Prefecture Seiwa Medical Center, Nara, Japan; 12 Department of Respiratory Medicine, Okinawa Chubu Hospital, Okinawa, Japan; 13 Department of Respiratory Medicine, Japanese Red Cross Medical Center, Tokyo, Japan; 14 Section of Education for Clinical Research, Kyoto University Hospital, Kyoto, Japan

**Keywords:** Idiopathic pulmonary fibrosis, Acute exacerbation, Corticosteroids

## Abstract

**Background:**

Although corticosteroid therapy with dose tapering is the most commonly used treatment for acute exacerbation of idiopathic pulmonary fibrosis (AE-IPF), there is no consensus on the tapering regimen. This study aimed to investigate the association between early corticosteroid dose tapering and in-hospital mortality in patients with AE-IPF.

**Methods:**

In this retrospective cohort study, we analyzed the data of a cohort from eight Japanese tertiary care hospitals and routinely collected administrative data from a cohort from 185 Japanese hospitals. Patients with AE-IPF were classified into the early and non-early tapering groups depending on whether the maintenance dose of corticosteroids was reduced within two weeks of admission. Propensity score analysis with inverse probability weighting (IPW) was performed to estimate the effect of early corticosteroid dose tapering.

**Results:**

The multi-center cohort included 153 eligible patients, of whom 47 (31%) died, whereas the administrative cohort included 229 patients, of whom 51 (22%) died. Patients with early tapering tended to have a better prognosis than those without it (unadjusted hazard ratio [95% confidence interval] 0.41 [0.22–0.76] and 0.65 [0.36–1.18] in the multi-center and administrative cohorts, respectively). After IPW, the early tapering group had a better prognosis than the non-early tapering group (IPW-adjusted hazard ratio [95% confidence interval] 0.37 [0.14–0.99] and 0.27 [0.094–0.83] in the multi-center and administrative cohorts, respectively).

**Conclusion:**

Early corticosteroid dose tapering was associated with a favorable prognosis in patients with AE-IPF. Further studies are warranted to confirm the effects of early corticosteroid dose tapering in patients with AE-IPF.

**Supplementary Information:**

The online version contains supplementary material available at 10.1186/s12931-022-02195-3.

## Introduction

Idiopathic pulmonary fibrosis (IPF), the most common idiopathic interstitial pneumonia (IP), is characterized by chronic progressive lung fibrosis. IPF has a poor prognosis, with which 5–15% of patients experience acute exacerbation (AE) every year, and AE-IPF has been associated with in-hospital mortality of 50–60%. [1, 2] Although several treatments for AE-IPF exist, [3, 4] none have been proven to be effective, and there is an urgent need for an effective treatment.

Corticosteroids are the most commonly used treatment for AE-IPF. Although no randomized controlled trials have investigated their efficacy for treating AE-IPF, their use has been weakly recommended in international guidelines, [5] and they are widely accepted as the standard treatment for AE-IPF. When corticosteroids are used to treat AE-IPF, their dose is often gradually tapered. [4]

However, there is no consensus on the regimen for corticosteroid dose tapering in patients with AE-IPF. Early tapering of corticosteroid dose has been found to reduce side effects without worsening prognosis in patients requiring systemic corticosteroids for treating other diseases. [6–9] However, the American Thoracic Society/European Respiratory Society/Japanese Respiratory Society/Latin American Thoracic Association guidelines for IPF do not specify any method for tapering steroids in patients with AE-IPF. [5] Furthermore, we believe that no study has been conducted on this topic in patients with AE-IPF. Therefore, there is a need to investigate the association between the steroid tapering method and prognosis in patients with AE-IPF.

This study aimed to investigate the relationship between early tapering of corticosteroid dose and short-term AE-IPF prognosis using data from two cohorts. We hypothesized that early tapering of corticosteroid dose will be associated with a better AE-IPF prognosis than non-early tapering.

## Methods

This study has been reported in accordance with the Strengthening the Reporting of Observational Studies in Epidemiology [10] and Reporting of studies Conducted using Observational Routinely collected health Data [11] statements (Table S1).

### Study design and data source

To verify the robustness of the results, this retrospective cohort study was conducted using data from two cohorts: a multi-center cohort from eight Japanese tertiary care hospitals and a Japanese administrative cohort. In the multi-center cohort, co-investigators and medical information department personnel at each institution confirmed data extracted directly from electronic medical records, and the principal researcher (KA) finalized the data. Distribution checks and logical checks were performed, and if there were outliers or suspicions, the principal researcher contacted each facility to inquire about them and corrected any errors. The administrative data used in this study are commercial database developed and maintained by the Health, Clinic, and Education Information Evaluation Institute, Kyoto, Japan, supported by Real World Data, Co., Ltd. (Kyoto, Japan). This database consists of the electronic medical records of approximately 20 million patients from 185 medical institutions across Japan but not from the eight hospitals from which the multi-center cohort was enrolled, and it contains information of both inpatients and outpatients on demographic characteristics, medications, procedures, disease names, and results of laboratory tests. The data were obtained through automatic extraction from the electronic medical records of each institution and anonymized using a peculiar patient identifier (https://www.hcei.or.jp/page/database). Sample types, units of test values, and actual test values were checked multiple times by full-time laboratory technicians to ensure reliability. Quality assurance was achieved by having the two researchers completely agree on the results obtained in this study from these data.

## Patients

### Multi-center cohort

From this cohort, patients aged over 40 years with disease names related to AE-IPF but not those related to secondary IP (e.g., chronic/fibrotic hypersensitivity pneumonitis and connective tissue disease [CTD]-associated interstitial lung disease [ILD]) and malignancy (Tables S2, S3) who were admitted to eight tertiary care hospitals between January 2016 and February 2019 were included. The detailed criteria have been described elsewhere. [12] In brief, we combined the International Classification of Diseases 10th Revision codes (e.g., J84.1, J84.9) and chart review including high-resolution computed tomography (HRCT) findings. The following 1 to 8 were defined as exclusion criteria: (1) secondary IP, (2) comorbid advanced cancer, (3) unilateral pneumonia, pulmonary embolism, or pneumothorax at admission, (4) refusal of treatment, and (5) no corticosteroid administration within 14 days of admission. We also excluded patients who (6) only received steroid pulse therapy, defined as > 500 mg/day of methylprednisolone equivalent, because we intended to investigate the effect of tapering the corticosteroid maintenance dose and (7) received only ≤ 10 mg/day of prednisolone equivalent throughout their hospitalization because such doses are often the same doses administered before hospitalization. Furthermore, we considered the immortal time, which is the period during which the outcome cannot occur in the cohort, as failure to account for this period can result in bias. [13] Clinically, corticosteroids for treating AE-IPF are often tapered after at least one week of admission, and tapering is unlikely to have been performed in patients who died within seven days of admission. This could cause immortal time bias against the non-early tapering group. To address this bias, we excluded (8) patients who died or were discharged within seven days of admission.

Eligibility in terms of AE-IPF diagnosis was determined by two pulmonologists based on the official American Thoracic Society/European Respiratory Society/Japanese Respiratory Society/Latin American Thoracic Association clinical practice guidelines for the diagnosis of IPF [14] and the diagnostic criteria for AE-IPF proposed by the international working group, [15] following which two expert radiologists evaluated the eligible patients’ HRCT findings. [12]

### Administrative cohort

From this cohort, patients aged over 40 years diagnosed with AE-IPF according to broad criteria (positive predictive value: 0.61, 95% confidence interval [CI]: 0.53–0.68) that were validated in a prior study were included. [12] We excluded patients who (1) did not receive corticosteroid therapy within 14 days of admission, (2) only received corticosteroid pulse therapy, (3) received only ≤ 10 mg/day of prednisolone equivalent throughout hospitalization, and (4) died or were discharged within seven days of admission.

### Exposure

Patients were classified into the early and non-early tapering groups according to the timing of corticosteroid dose tapering. The Japanese guidelines for the treatment of IPF contain the following statement regarding corticosteroids at the time of acute exacerbation: “The regimen in Japan frequently consists of corticosteroid pulse therapy at 1 g/day for 3 days (repeated 1–4 times at weekly intervals while observing reaction) and subsequent corticosteroid treatment maintained at 0.5–1 mg/kg, with dose reduction every 2–4 weeks by 5 mg at a time depending on patient condition.” For example, in a patient weighing 50 kg receiving 1 mg/kg of steroids, a subsequent 5 mg dose reduction signifies a 10% tapering. Based on this guideline statement, early tapering was defined as a reduction in corticosteroid maintenance dose of > 10% within two weeks of admission (excluding reduction after steroid pulse therapy), even if the dose was increased during the subsequent hospitalization. Steroid dose was defined based on the dose of prednisolone equivalent administered initially or after steroid pulse therapy as follows: high dose, ≥ 1.0 mg/kg/day; moderate dose, 0.5 − 1.0 mg/kg/day, and low dose, < 0.5 mg/kg/day. For patients whose body weight data were not available, the steroid dose was defined as follows: high dose, ≥ 50 mg/day of prednisolone equivalent; moderate dose, 25 − 50 mg/day; and low dose, < 25 mg/day. Patients were considered to have received steroid pulse therapy if they received it before the seventh day of hospitalization. For steroid doses other than pulse therapy, we considered the first dose started within seven days after admission as the initial dose.

### Outcome

The primary outcome of this study was the time to all-cause in-hospital mortality within 90 days. Patients were followed until discharge or 90 days.

### Data collection

Data regarding the patients’ baseline clinical characteristics, blood test results, HRCT findings, as well as the treatment before and after admission were collected. We also retrieved data regarding the blood and imaging findings on day 7 (± 3) of admission. Some of these data (e.g., imaging data) were only available for the multi-center cohort. The modified HRCT score, which is a simplified version of the original HRCT score, [16, 17] was recorded as a semiquantitative assessment of fibroproliferative changes (Appendix S1). Two independent chest radiologists (TJ and KF) with 33 and 34 years of experience, respectively, who were blinded to the patients’ conditions determined the modified HRCT score, and the mean score was used. Charlson Comorbidity Index was used as an indicator of comorbidity. [18] We considered treatment (e.g., immunosuppressant agents) initiated by the seventh day of admission as the treatment received. The confounding variables considered have been described in detail in Appendix S1.

### Sample size

Since AE-IPF is a rare condition and data on it is difficult to collect, we did not perform sample size calculation but aimed to collect data on as many cases as possible.

### Statistical analyses

The patients’ baseline characteristics are presented as median (interquartile range) for continuous variables and frequency (%) for categorical variables. Intraclass correlation coefficients and Bland–Altman plots were used to assess the agreement between the modified HRCT scores by the two radiologists. [19] Missing data were imputed by performing multiple imputations (Appendix S1). Propensity score analysis with inverse probability weighting (IPW) was performed to adjust for potential confounders (Appendix S1). We calculated the propensity score weight for steroid dose tapering using logistic regression with predetermined potential confounders. Stabilized weights were used to reduce the effects of extreme values of the estimated propensity scores. We evaluated the balance of confounding variables between the two groups using absolute values of standardized differences and kernel density plots, and a standardized difference of < 0.25 was regarded as balanced. [20] Unadjusted and IPW-adjusted survival curves were constructed for each group in each cohort using the Kaplan–Meier method. We performed the main analysis using the IPW-weighted Cox proportional hazard model to estimate the hazard ratio (HR). Robust variance estimation was used to consider the cluster effect at the institution level in the multi-center cohort.

### Sensitivity analyses

A sensitivity analysis was performed using IPW after excluding patients who died or were discharged within 10 days of admission. We also performed a sensitivity analysis to assess an HR using a multivariable Cox proportional hazard model without IPW weighting. In the multivariable Cox analysis, we adjusted for the following confounding variables: age, sex, respiratory status, imaging findings, CCI score, and blood test findings for the multi-center cohort, and age, sex, CCI score, and blood test findings for the administrative cohort. We also performed multivariable Cox analysis in the multi-center cohort by adjusting for the same variables as in the administrative cohort. Among the blood test findings, LDH level at admission and on day 7 (± 3) of admission, CRP level at admission and on day 7 (± 3) of admission, KL-6 level at admission, and albumin level at admission and on day 7 (± 3) of admission were prioritized, in that order. All statistical analyses were performed using STATA/SE version 16.0 (Stata Corp., College Station, TX, USA).

## Results

### Patients’ characteristics

**Figure 1:**
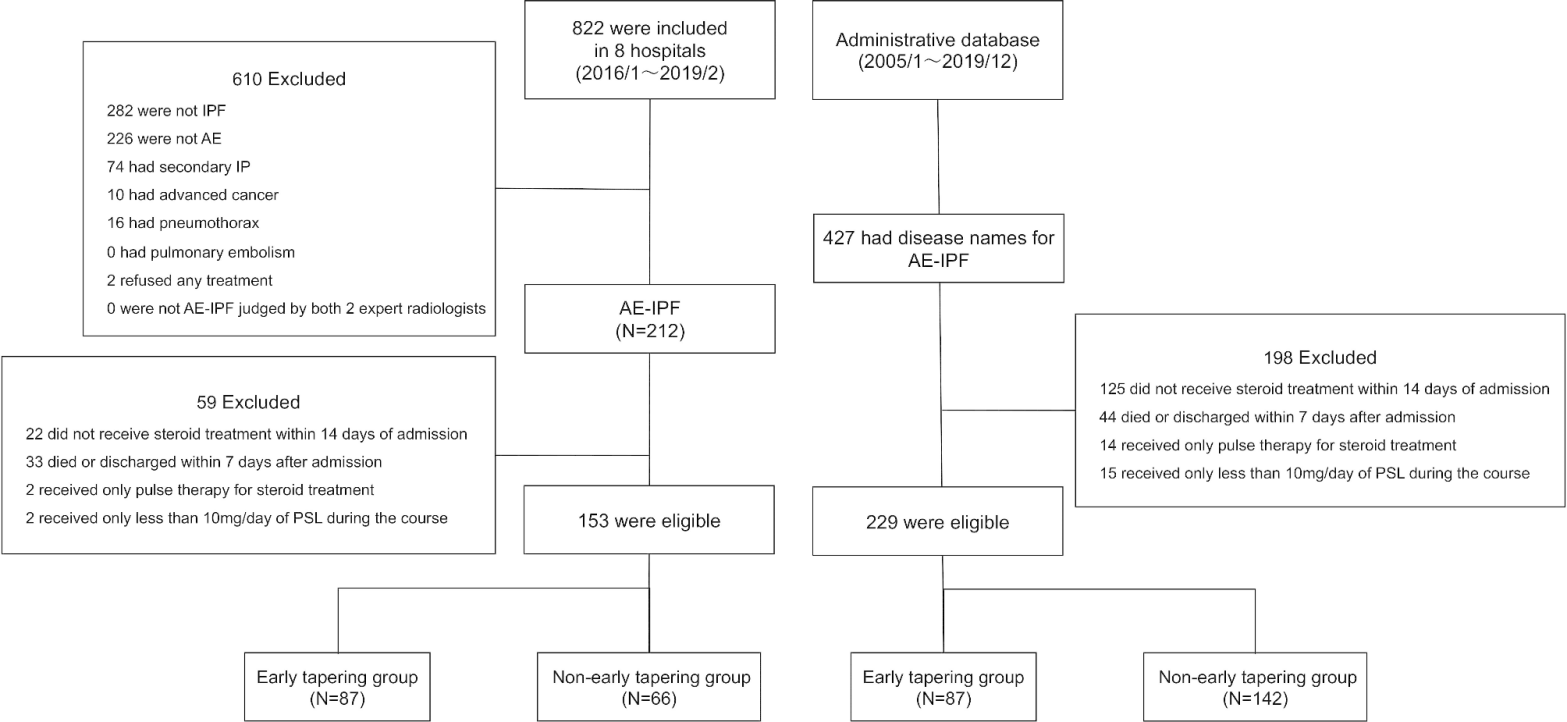
Flow chart of patient enrolment. AE, acute exacerbation; IPF, idiopathic pulmonary fibrosis; IP, interstitial pneumonia; PSL; prednisolone

The flowchart of patient enrolment is shown in Fig. 1. There were 153 (87 and 66 in the early and non-early tapering groups, respectively) and 229 (87 and 142 in the early and non-early tapering groups, respectively) eligible patients in the multi-center and administrative cohorts, respectively. The median follow-up periods were 23 days (interquartile range, 16 − 30 days) and 25 days (16 − 42 days) in the multi-center and administrative cohorts, respectively. All patients were followed up until death, discharge, or 90 days of hospitalization. The baseline characteristics of the patients are shown in Table 1, and the details of the treatment after hospitalization are shown in Table 2. Bland–Altman plots of the comparison of HRCT scores between the two radiologists showed no obvious proportional or fixed bias (Figure S1), and the intraclass correlation coefficients (0.45; 95% CI 0.28–0.59) indicated a fair association between the scores by the two radiologists. During the course of the study, 47 (31%) and 51 (22%) patients in the multi-center cohort and administrative cohort died, respectively.

**Table 1:** Baseline characteristics

	Multi-center cohort data			Administrative cohort data		
Variable	Overall (n = 153)	Early tapering group (n = 87)	Non-early tapering group (n = 66)	Overall (n = 229)	Early tapering group (n = 87)	Non-early tapering group (n = 142)
Etiology of AE (idiopathic/ infection/ drug/ aspiration)	130(85%)/ 16(10%)/ 6(4%)/ 1(1%)	74(85%)/ 8(9%)/ 5(5%)/ 0(0%)	56(85%)/ 8(12%)/ 1(2%)/ 1(2%)	NA	NA	NA
Institution (A/ B/ C/ D/ E/ F/ G/ H)	59(39%)/ 35(23%)/ 26(17%)/ 19(12%)/ 5(3%)/ 5(3%)/ 2(1%)/ 2(1%)	44(51%)/ 7(8%)/ 21(24%)/ 7(8%)/ 3(3%)/ 3(3%)/ 2(2%)/ 0(0%)	15(23%)/ 28(42%)/ 5(8%)/ 12(18%)/ 2(3%)/ 2(3%)/ 0(0%)/ 2(3%)	NA	NA	NA
Age (years)	78 (73–84)	78 (71–82)	78 (73–84)	77 (71–82)	78 (72–82)	77 (70–82)
Sex (male)	113 (74%)	70 (80%)	43 (65%)	160 (70%)	68 (78%)	92 (65%)
KL-6 level (U/mL)	1278 (780–2080)	1262 (735–1950)	1305 (811–2275)	1118 (684–1814)	1043 (676–1566)	1152 (711–1873)
Missing data	18 (12%)	7 (8%)	11 (17%)	49 (21%)	14 (16%)	35 (25%)
LDH level (IU/L)	344 (278–437)	329 (263–405)	374 (301–442)	320 (260–389)	320 (242–399)	320 (267–389)
Missing data	2 (1%)	2 (2%)	0 (0%)	12 (5%)	5 (6%)	7 (5%)
CRP level (mg/dL)	6.5 (3.2–11.6)	6.6 (3.6–11.7)	5.5 (2.6–10.3)	4.8 (1.5–11.1)	5.8 (1.6–11.8)	4.4 (1.2–10.6)
Missing data	3 (2%)	2 (2%)	1 (2%)	12 (5%)	5 (6%)	7 (5%)
Albumin level (g/dL)	3.1 (2.8–3.4)	3.1 (2.7–3.5)	3.1 (2.8–3.3)	3.2 (2.9–3.5)	3.1 (2.8–3.5)	3.2 (2.9–3.5)
Missing data	4 (3%)	3 (3%)	1 (2%)	16 (7%)	6 (7%)	10 (7%)
PaO_2_/FiO_2_	186 (101–284)	223 (90–295)	178 (115–268)	NA	NA	NA
Missing data	37 (24%)	27 (31%)	10 (15%)			
CCI ( ≦ 2/3 or 4/≧5)	116 (76%)/ 29 (19%)/8 (5%)	63 (72%)/ 19 (22%)/ 5 (6%)	53 (80%)/ 10 (15%)/ 3 (5%)	76 (39%)/ 60 (30%)/ 61 (31%)	29 (39%)/ 28 (38%)/ 17 (23%)	47 (38%)/ 32 (26%)/ 44 (36%)
Missing data	0 (%)	0 (%)	0 (%)	32 (14%)	13 (15%)	19 (13%)
LDH level on day 7 (± 3) of admission (IU/L)	306 (251–427)	286 (219–369)	350 (261–447)	274 (218–344)	267 (203–316)	279 (225–361)
Missing data	7 (5%)	5 (6%)	2 (3%)	43 (19%)	28 (32%)	15 (11%)
CRP level on day 7 (± 3) of admission (mg/dL)	1.3 (0.5–4.3)	1.1 (0.4–2.7)	2.1 (0.7–5.3)	1.0 (0.3–3.2)	1.0 (0.3–4.1)	1.0 (0.3–3.2)
Missing data	6 (4%)	4 (5%)	2 (3%)	40 (17%)	28 (32%)	12 (8%)
Albumin level on day 7 (± 3) of admission (g/dL)	2.7 (2.4–3.2)	2.8 (2.5–3.2)	2.7 (2.3–3.1)	2.9 (2.5–3.2)	2.8 (2.3-3.0)	2.9 (2.6–3.2)
Missing data	29 (19%)	18 (21%)	11 (17%)	72 (31%)	37 (43%)	35 (25%)
SpO_2_/FiO_2_ on day 7 (± 3) of admission	306 (207–400)	323 (242–422)	272 (160–392)	NA	NA	NA
Missing data	6 (4%)	5 (6%)	1 (2%)			
HRCT score	260 (225–295)	260 (220–305)	270 (230–295)	NA	NA	NA
Changes in Xp or CT findings on day7 (± 3) of admission (improve/stable/worsening)	85 (59%)/ 39 (27%)/ 21 (14%)	60 (74%)/15 (19%)/6 (7%)	25 (39%)/24 (38%)/15 (23%)	NA	NA	NA
Missing data	8 (5%)	6 (7%)	2 (3%)			
Pre-admission treatment (No missing data)						
Oral steroid	46 (30%)	18 (21%)	28 (42%)	65 (28%)	19 (22%)	46 (32%)
Immunosuppressant	18 (12%)	10 (11%)	8 (12%)	14 (6%)	7 (8%)	7 (5%)
Pirfenidone	12 (8%)	8 (9%)	4 (6%)	25 (11%)	9 (10%)	16 (11%)
Nintedanib	13 (8%)	6 (7%)	7 (11%)	4 (2%)	2 (2%)	2 (1%)
Macrolide	24 (16%)	19 (22%)	5 (8%)	12 (5%)	4 (5%)	8 (6%)
ST	40 (26%)	23 (26%)	17 (26%)	29 (13%)	9 (10%)	20 (14%)
LTOT	38 (25%)	15 (17%)	23 (35%)	NA	NA	NA
In hospital mortality	47 (31%)	15 (17%)	32 (48%)	51 (22%)	16 (18%)	35 (25%)

AE, acute exacerbation; CCI, Charlson Comorbidity Index; CRP, C reactive protein; CT, computed tomography; FiO_2_, fraction of inspired oxygen; HRCT, high-resolution CT; LDH, lactate dehydrogenase; LTOT, long-term oxygen therapy; NA, not available; SpO_2_, peripheral oxygen saturation; ST, trimethoprim-sulfamethoxazole; Xp, X-ray photography; KL-6, Krebs von den Lugen-6

Data are shown as the number (percentage) or median (interquartile range)

**Table 2:** Treatment information during hospitalization (no missing data)

	Multi-center cohort data			Administrative cohort data		
Variable	Overall (n = 153)	Early tapering group (n = 87)	Non-early tapering group (n = 66)	Overall (n = 229)	Early tapering group (n = 87)	Non-early tapering group (n = 142)
Steroid pulse therapy	119 (78%)	70 (80%)	49 (74%)	117 (51%)	54 (62%)	63 (44%)
Dose of steroid (low/moderate/high)	26 (17%)/ 66 (43%)/ 61 (40%)	9 (10%)/38 (44%)/ 40 (46%)	17 (26%)/28 (42%)/ 21 (32%)	73 (32%)/ 68 (30%) / 88 (38%)	10 (11%) / 27 (31%)/ 50 (57%)	63 (44%)/ 41 (29%)/ 38 (27%)
Immunosuppressant	22 (14%)	9 (10%)	13 (20%)	31 (14%)	17 (20%)	14 (10%)
β-lactam combination	90 (59%)	52 (60%)	38 (58%)	114 (50%)	45 (52%)	69 (49%)
Macrolide	81 (53%)	49 (56%)	32 (48%)	16 (7%)	8 (9%)	8 (6%)
Fluoroquinolone	4 (3%)	4 (5%)	0 (0%)	39 (17%)	16 (18%)	23 (16%)
ST	69 (45%)	41 (47%)	28 (42%)	98 (43%)	36 (41%)	62 (44%)
rTM	5 (3%)	5 (6%)	0 (0%)	1 (0%)	0 (0%)	1 (1%)
PMX-DHP	0 (0%)	0 (0%)	0 (0%)	0 (0%)	0 (0%)	0 (0%)
Mechanical ventilation	43 (28%)	22 (25%)	21 (32%)	6 (3%)	3 (3%)	3 (2%)
IPPV	12 (8%)	7 (8%)	5 (8%)	NA	NA	NA
NPPV	34 (22%)	17 (20%)	17 (26%)	NA	NA	NA
HFNC	42 (27%)	27 (31%)	15 (23%)	NA	NA	NA
DNI	89 (58%)	42 (48%)	47 (71%)	NA	NA	NA

DNI, do not intubate; HFNC, high-flow nasal cannula; IPPV, invasive positive pressure ventilation; NA, not available; NPPV, non-invasive positive pressure ventilation; PMX-DHP, polymyxin B-immobilized fiber direct hemoperfusion; rTM, recombinant thrombomodulin; ST, trimethoprim-sulfamethoxazole

Data are shown as number (percentage)

### Association between early tapering of corticosteroid dose and mortality

Distributions of the IPW-adjusted propensity score of the early and non-early tapering groups were well balanced (Figures S2, S3), with all standardized differences being less than 25% (Figure S4).

**Figure 2:**
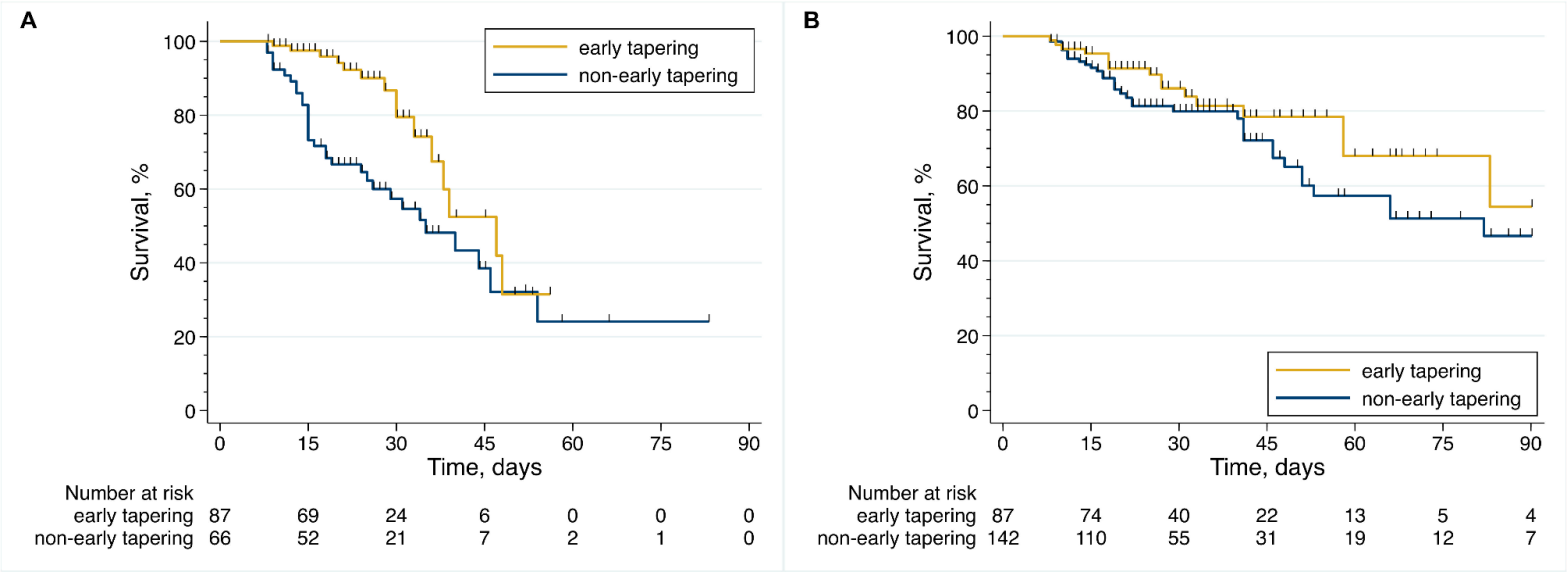
Kaplan–Meier curves of AE-IPF patients with early or non-early tapering of corticosteroid dose. (**A**) Multi-center cohort. (**B**) Administrative cohort. AE-IPF, acute exacerbation of idiopathic pulmonary fibrosis

Kaplan–Meier curves (unadjusted HR [95% CI] 0.41 [0.22–0.76] in the multi-center cohort and 0.65 [0.36–1.18] in the administrative cohort;Fig. 2 A, B) and the IPW-adjusted survival analysis (IPW-adjusted HR [95% CI] 0.37 [0.14–0.99] in the multi-center cohort and 0.27 [0.094–0.83] in the administrative cohort; Table 3) showed that the early tapering group had a better prognosis than the non-early tapering group in both cohorts. The IPW-adjusted Kaplan–Meier curves are shown inFig. 3 A, B.

**Figure 3:**
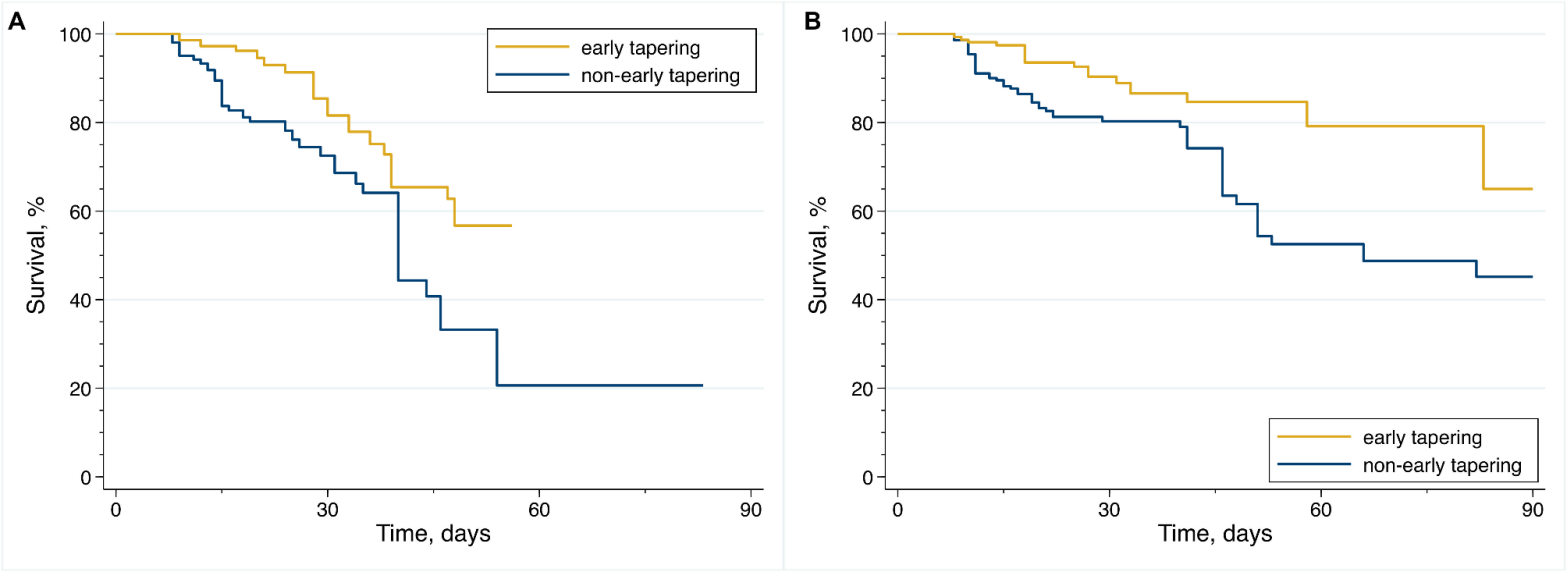
IPW–adjusted Kaplan–Meier curves of AE-IPF patients with early or non-early corticosteroid dose tapering. (**A**) Multi-center cohort. (**B**) Administrative cohort. AE-IPF, acute exacerbation of idiopathic pulmonary fibrosis; IPW, inverse probability weighting

**Table 3:** Results of univariate, multivariate, and IPW-adjusted survival analyses for in-hospital mortality in patients with early or non-early tapering steroid group

	Multi-center cohort data		Administrative cohort data	
	crude HR (95% CI)	adjusted HR (95% CI)	crude HR (95% CI)	adjusted HR (95% CI)
Univariate analysis	0.41 (0.22, 0.76)	−	0.65 (0.36, 1.18)	−
Main analysis (IPW-adjusted survival analysis)	−	0.37 (0.14, 0.99) *	−	0.27 (0.094, 0.83) †
Sensitivity analysis 1 (IPW-adjusted survival analysis) ††	−	0.28 (0.078, 0.98) *	−	0.35 (0.12, 1.04) †
Sensitivity analysis 2 (Cox proportional hazard model)	−	0.36 (0.18, 0.72) ¶	−	−
Sensitivity analysis 3 (Cox proportional hazard model)	−	0.41 (0.19, 0.87) §	−	0.59 (0.31, 1.13) §

* IPW was calculated using a logistic regression model that includes age, sex, LDH level, CRP level, albumin level, KL-6 level, CCI ( ≦ 2, 3 or 4, ≧5), LDH level on day 7 (± 3) of admission, CRP level on day 7 (± 3) of admission, albumin level on day 7 (± 3) of admission, LTOT before AE (yes, no), PaO_2_/FiO_2_, SpO_2_/FiO_2_ on day 7 (± 3) of admission, HRCT score, change in imaging findings at day 7 (± 3) of admission (improvement, stable or worsening), steroid administration before admission (yes, no), immunosuppressant administration before admission (yes, no), steroid pulse therapy (yes, no), initial steroid dose (low, moderate, high), and immunosuppressant after admission (yes, no)

† IPW was calculated using a logistic regression model that includes age, sex, LDH level, CRP level, albumin level, KL-6 level, CCI ( ≦ 2, 3 or 4, ≧5), LDH level on day 7 (± 3) of admission, CRP level on day 7 (± 3) of admission, albumin level on day 7 (± 3) of admission, steroid administration before admission (yes, no), immunosuppressant administration before admission (yes, no), steroid pulse therapy (yes, no), initial steroid dose (low, moderate, high), and immunosuppressant after admission (yes, no)

†† After excluding patients who died or were discharged within 10 days after admission

¶ Adjusted for age, sex, LTOT before AE (yes, no), HRCT score, PaO_2_/FiO_2_, SpO_2_/FiO_2_ on day 7 (± 3) of admission, and changes in imaging findings on day 7 (± 3) of admission (improve, stable or worsening)

§ Adjusted for age, sex, LDH level, CRP level, CCI ( ≦ 2, 3 or 4, ≧5), LDH level on day 7(± 3) of admission, and CRP on day 7 (± 3) of admission

AE, acute exacerbation; CCI, Charlson Comorbidity Index; CRP, C reactive protein; CI, confidence interval; FiO_2_, fraction of inspired oxygen; HR, hazard ratio; HRCT, high-resolution computed tomography; IPW, inverse probability weighting; KL-6, Krebs von den Lugen-6; LDH, lactate dehydrogenase; LTOT, long-term oxygen therapy; PaO_2_, arterial oxygen tension; SpO_2_, peripheral oxygen saturation

### Sensitivity analyses

Sensitivity analysis performed using IPW by excluding patients who had died or been discharged within 10 days of admission showed that the early tapering group tended to have a better prognosis than the non-early tapering group (IPW-adjusted HR [95% CI]: 0.28 [0.078–0.98] in the multi-center cohort and 0.35 [0.12–1.04] in the administrative cohort; Table 3, Figures S5 A, B). Sensitivity analysis of the multi-center cohort data performed using the Cox proportional hazard model and adjusting for age, sex, imaging findings (HRCT score and change in imaging findings on day 7 [± 3] of admission), and respiratory status (long-term oxygen therapy use before AE, arterial oxygen tension (PaO_2_)/fraction of inspired oxygen (FiO_2_), and peripheral oxygen saturation (SpO_2_)/FiO_2_ on day 7 [± 3] of admission) showed that the early tapering group had a better prognosis than the non-early tapering group (adjusted HR [95% CI] 0.36 [0.18–0.72]; Table 3). Multivariate analysis with adjustment for age, sex, serum lactate dehydrogenase level at admission and on day 7 (± 3) of admission, serum C-reactive protein level at admission and on day 7 (± 3) of admission, as well as the Charlson Comorbidity Index (Appendix S1) showed that patients in the early tapering group had a better prognosis than those in the non-early tapering group (adjusted HR [95% CI] 0.41 [0.19–0.87] in the multi-center cohort and 0.59 [0.31–1.13] in the administrative cohort; Table 3).

## Discussion

Our study investigated the association between early tapering of corticosteroid dose and mortality in patients with AE-IPF from two cohorts. We found that early corticosteroid dose tapering was associated with a favorable prognosis in both cohorts. Although the sensitivity analyses revealed wider 95% CIs, the direction of the point estimates was consistent.

The results of our study suggest that it may be beneficial to taper the corticosteroid dose early in patients with AE-IPF. We believe our study is novel because no previous study has examined the timing of corticosteroid tapering in patients with AE-IPF. A retrospective single-center cohort study reported that there was an association between higher average daily steroid dosage and higher in-hospital mortality in patients with AE-IPF, [21] consistent with the results of our study. We believe the results of our study are more robust because we adjusted for more important confounding variables with a larger sample size. Meanwhile, another retrospective single-center cohort study reported that a high total corticosteroid dose in the first 30 days was associated with fewer relapses of AE-IPF than a low total dose [22]; these results appear to contradict those of our study. However, that study had several limitations, such as the lack of sufficient adjustment for confounding factors (e.g., PaO_2_/FiO_2_, HRCT findings, and blood test results), exclusion of patients who died during hospitalization, and lack of evaluation of the association with mortality. Studies investigating the harmful effects of high-dose corticosteroids should include mortality as an outcome, as was done in our study. Retrospective cohort studies have shown that a high initial corticosteroid dose is associated with a good prognosis in patients with AE- idiopathic IP or AE-ILD. [23, 24] As in those studies, the early tapering group in this study received a higher initial steroid dose than the non-early tapering group. However, in our study, the early tapering group had a better prognosis than the non-early tapering group even after adjusting for the initial dose. Consequently, both the initial use of high-dose corticosteroids and early tapering of corticosteroid dose might be associated with improved outcomes.

In this study, we showed that early tapering of corticosteroid dose is associated with a favorable prognosis. This may be biologically plausible because viral infection has been reported to be a common cause of AE-IPF, [15] and the use of corticosteroids has been reported to delay viral excretion. [25] In addition, long-term use of high doses of steroids increases the frequency of serious complications. [26]

This study has two research implications. First, this was an observational study of two cohorts in which we examined the association between early tapering of corticosteroid dose and prognosis in patients with AE-IPF. A randomized controlled trial is needed to confirm our results. Second, we excluded patients with AE of non-specific IP (NSIP) or secondary IPs such as CTD-ILD or chronic/fibrotic hypersensitivity pneumonitis. However, corticosteroids and immunosuppressive drugs are widely used in the treatment of CTD-ILD of both acute and chronic onset, [27] and these treatments may be more effective for AE of NSIP or secondary IP than AE-IPF. The feasibility of early tapering of corticosteroid dose in patients with AE of NSIP or secondary IP requires further investigation.

Our study has several strengths. First, we assessed the relationship between early tapering of corticosteroid dose and prognosis of patients with AE-IPF using data from two cohorts, and similar results were obtained with both datasets. In addition, we performed sensitivity analyses to confirm the robustness of our results. Second, the sample size of both cohorts included in our study was larger than that of previous studies of patients with AE-IPF, and multiple potential confounding variables were adjusted. In particular, in addition to baseline characteristics, we adjusted HRCT scores, which were determined by two expert radiologists, and several factors on day 7 of admission, such as blood test data, respiratory status, and changes in imaging.

This study has several limitations. First, misclassification cannot be completely ruled out because of the difficulty in diagnosing this condition. However, efforts to reduce misclassification in the multi-center cohort were made by the two chest radiologists who interpreted the HRCT findings. Second, the algorithm used to extract patients from the administrative cohort has a positive predictive value of only approximately 60%. [12] Therefore, some patients without true AE-IPF might have been included. In fact, the administrative cohort had a lower mortality than the multi-center cohort, suggesting that it may have included a less severely ill population. However, the variability in patient characteristics between the early and non-early tapering groups was not notably different between the two cohorts, suggesting a low risk of bias that could impact the results. Third, due to limitations of the dataset of the administrative cohort, there were multiple unadjusted confounding variables. To show that our results are robust, we separately evaluated data from the multi-center cohort, which included information on imaging findings and respiratory status. Fourth, this is an observational study, and confounding by indication for treatment cannot be completely eliminated. However, we adjusted for most of the previously reported important prognostic factors at baseline. In addition, we adjusted for confounding variables at 7 days after admission. Since changes in each finding 7 days after admission could influence the decision to taper the corticosteroid dose, we believe these adjustments reduced confounding by indication for treatment. Therefore, we believe that we have provided as much evidence as possible at this time. Fifth, since patients who did not receive corticosteroids were excluded from this study, we cannot discuss whether corticosteroids should be used for treating AE-IPF, and further studies on this topic are needed. Sixth, in this study, we defined corticosteroid dose tapering within two weeks of admission as early tapering. However, we did not account for subsequent complex tapering. The method of subsequent steroid administration was more complex, with the patterns varying from case to case. The relationship between detailed steroid administration methods and the prognosis is unknown. Future randomized controlled trials with protocolized methods of corticosteroid tapering are needed. Finally, the prognosis of patients after discharge or transfer is unknown. We will conduct another study to evaluate long-term outcomes such as 90-day mortality using a multi-center cohort.

In conclusion, early tapering of corticosteroid dose was associated with a lower risk of in-hospital mortality in patients with AE-IPF. Our results suggest that physicians can consider the practice of tapering the corticosteroid dose early in patients with AE-IPF. Further randomized controlled trials are warranted to investigate the effect of early tapering of corticosteroid dose on outcomes in patients with AE-IPF.

## Electronic supplementary material

Below is the link to the electronic supplementary material.

Supplementary Material 1

Supplementary Material 2

Supplementary Material 3

Supplementary Material 4

Supplementary Material 5

Supplementary Material 6

Supplementary Material 7

Supplementary Material 8

Supplementary Material 9

Supplementary Material 10

## Data Availability

The data that support the findings of this study are available on request from the corresponding author upon reasonable request. The data are not publicly available due to privacy or ethical restrictions.

## List of abbreviations

AE: acute exacerbation.
CI: confidence interval.
CTD: connective tissue disease.
FiO_2_: fraction of inspired oxygen.
HR: hazard ratio.
HRCT: high-resolution computed tomography.
IP: interstitial pneumonia.
ILD: interstitial lung disease.
IPF: idiopathic pulmonary fibrosis.
IPW: inverse probability weighing.
NSIP: non-specific IP.
PaO_2_: arterial oxygen tension.
SpO_2_: peripheral oxygen saturation.
