# Correction: Early corticosteroid dose tapering in patients with acute exacerbation of idiopathic pulmonary fibrosis

**DOI:** 10.1186/s12931-023-02411-8

**Published:** 2023-04-10

**Authors:** Keisuke Anan, Yuki Kataoka, Kazuya Ichikado, Kodai Kawamura, Takeshi Johkoh, Kiminori Fujimoto, Kazunori Tobino, Ryo Tachikawa, Hiroyuki Ito, Takahito Nakamura, Tomoo Kishaba, Minoru Inomata, Tsukasa Kamitani, Hajime Yamazaki, Yusuke Ogawa, Yosuke Yamamoto

**Affiliations:** 1 Department of Healthcare Epidemiology, Graduate School of Medicine, Kyoto University, Yoshida Konoe-cho Sakyo-ku, Kyoto-City, 606-8501 Japan; 2 Division of Respiratory Medicine, Saiseikai Kumamoto Hospital, Kumamoto, Japan; 3 Systematic Review Workshop Peer Support Group (SRWS-PSG), Osaka, Japan; 4 Department of Internal Medicine, Kyoto Min-Iren Asukai Hospital, Kyoto, Japan; 5 Section of Clinical Epidemiology, Department of Community Medicine, Graduate School of Medicine, Kyoto University, Kyoto, Japan; 6 Department of Radiology, Kansai Rosai Hospital, Hyogo, Japan; 7 Department of Radiology, Kurume University School of Medicine, Fukuoka, Japan; 8 Department of Respiratory Medicine, Iizuka Hospital, Fukuoka, Japan; 9 Department of Respiratory Medicine, Kobe City Medical Center General Hospital, Hyogo, Japan; 10 Department of Pulmonology, Kameda Medical Center, Chiba, Japan; 11 Department of General Internal Medicine, Nara Prefecture Seiwa Medical Center, Nara, Japan; 12 Department of Respiratory Medicine, Okinawa Chubu Hospital, Okinawa, Japan; 13 Department of Respiratory Medicine, Japanese Red Cross Medical Center, Tokyo, Japan; 14 Section of Education for Clinical Research, Kyoto University Hospital, Kyoto, Japan

**Correction** **:** **Respiratory Research (2022) 23:291** 10.1186/s12931-022-02195-3

Following the publication of the original article [1], it was noted that the additional files 8, 9, and 10 were processed incorrectly due to miscommunication during the correction process. It has been corrected in this correction

The original article has been corrected.

## Supplementary Information

**Additional file 8: Table S1. **The RECORD statement—checklist of items, extended from the STROBE statement, that should be reported in observational studies using routinely collected health data.

**Additional file 9: Table S2.** Disease names adopted as inclusion criteria.

**Additional file 10: Table S3. **Disease names adopted as exclusion criteria.
