# Supplementary material for: Early corticosteroid dose tapering in patients with acute exacerbation of idiopathic pulmonary fibrosis
Source: Respir Res. 2022 Oct 26;23:291. doi: 10.1186/s12931-022-02195-3 (PMC9609246; doi:10.1186/s12931-022-02195-3)
Supplement: Supplementary file 6 — Supplementary Material 6 [file 12931_2022_2195_MOESM6_ESM.docx]

**Figure Legends for Supplementary Figures**

Figure S1. Bland–Altman plot of the participants’ HRCT scores. The dashed lines show the mean values of all differences between the two observers, and the dotted line represents the 95% limit of agreement. HRCT: high-resolution computed tomography

Figure S2. Kernel density plots representing the distribution of propensity scores in the early and non-early tapering groups of the multi-centre cohort before (A) and after (B) inverse probability weighting.

Figure S3. Kernel density plots representing the distribution of propensity scores in the early and non-early tapering groups of the administrative cohort before (A) and after (B) inverse probability weighting.

Figure S4. Covariate balance before and after inverse probability weighting. (A) Multi-centre cohort. (B) Administrative cohort.

Figure S5. IPW–adjusted Kaplan–Meier curves of AE-IPF patients with early or non-early corticosteroid dose tapering. Patients who died or were discharged within 10 days of admission were excluded. (A) Multi-center cohort. (B) Administrative cohort.

AE-IPF, acute exacerbation of idiopathic pulmonary fibrosis; IPW, inverse probability weighting
