# Supplementary material for: Early corticosteroid dose tapering in patients with acute exacerbation of idiopathic pulmonary fibrosis
Source: Respir Res. 2022 Oct 26;23:291. doi: 10.1186/s12931-022-02195-3 (PMC9609246; doi:10.1186/s12931-022-02195-3)
Supplement: Supplementary file 7 — Supplementary Material 7 [file 12931_2022_2195_MOESM7_ESM.docx]

**Appendix S1**

**Modified high-resolution computed tomography score**

To calculate the modified high-resolution computed tomography (HRCT) score, based on previous reports,^1,2^ HRCT findings were first graded on a scale of 1 to 6 using a classification system that correlates with histopathological findings as follows: score 1, normal attenuation; score 2, ground-glass attenuation; score 3, consolidation; score 4, ground-glass attenuation with traction bronchiolectasis or bronchiectasis; score 5, consolidation with traction bronchiolectasis or bronchiectasis; and score 6, honeycombing. Next, we estimated the range of each abnormality by visually assessing the percentage of the lung parenchyma that was involved (in 10% increments). Finally, the percentage area was multiplied by a score of 1–6, and the six scores were summated to calculate the overall score for each patient.

**Confounding variables**

Based on prior research, clinical experience, and data availability,^3-5^ we considered the following variables as potential confounding variables a priori: age, sex, long-term oxygen therapy use before acute exacerbation, arterial oxygen tension (PaO_2_)/fraction of inspired oxygen (FiO_2_), peripheral oxygen saturation (SpO_2_)/FiO_2_ on day 7 (±3) of admission, serum lactate dehydrogenase (LDH) level at admission and on day 7 (±3) of admission, serum C-reactive protein (CRP) level at admission and on day 7 (±3) of admission, serum albumin level at admission and on day 7 (±3) of admission, serum KL-6 level at admission, Charlson Comorbidity Index (CCI) score, HRCT score, change in imaging findings on day 7 (±3) of admission, steroid use before admission, immunosuppressant use before and after admission, use of steroid pulse therapy, and dose of steroid therapy (low/moderate/high). For the early tapering group, data obtained before or on the day of tapering were considered the day 7 (±3) of admission data, and data after tapering were not used.

**Method of imputation of missing data**

We performed multivariate imputation using chained equations to impute the missing data. We constructed the imputation model using the variables used to calculate the propensity score, along with an institute variable (multi-center cohort) and the Nelson-Aalen cumulative hazards estimate.^6^ Twenty imputed datasets were created, and the results were pooled using Rubin’s rules.^7^

**References**

1. Ichikado K, Suga M, Muranaka H, Gushima Y, Miyakawa H, Tsubamoto M, et al. Prediction of prognosis for acute respiratory distress syndrome with thin-section CT: validation in 44 cases. Radiology. 2006;238:321-9.

2. Ichikado K, Muranaka H, Gushima Y, Kotani T, Nader HM, Fujimoto K, et al. Fibroproliferative changes on high-resolution CT in the acute respiratory distress syndrome predict mortality and ventilator dependency: a prospective observational cohort study. BMJ Open. 2012;2:e000545.

3. Kishaba T, Tamaki H, Shimaoka Y, Fukuyama H, Yamashiro S. Staging of acute exacerbation in patients with idiopathic pulmonary fibrosis. Lung. 2014;192:141-~~14~~9.

4. Kang HS, Cho KW, Kwon SS, Kim YH. Prognostic significance of Glasgow prognostic score in patients with acute exacerbation of idiopathic pulmonary fibrosis. Respirology. 2018;23:206-12.

5. Fujimoto K, Taniguchi H, Johkoh T, Kondoh Y, Ichikado K, Sumikawa H, et al. Acute exacerbation of idiopathic pulmonary fibrosis: high-resolution CT scores predict mortality. Eur Radiol. 2012;22:83-92.

6. White IR, Royston P. Imputing missing covariate values for the Cox model. Stat Med. 2009;28:1982-98.

7. Rubin DB, Schenker N. Multiple imputation in health-care databases: an overview and some applications. Stat Med. 1991;10:585-98.
