# Supplementary material for: Correction: Early corticosteroid dose tapering in patients with acute exacerbation of idiopathic pulmonary fibrosis
Source: Respir Res. 2023 Apr 10;24:107. doi: 10.1186/s12931-023-02411-8 (PMC10084659; doi:10.1186/s12931-023-02411-8)
Supplement: Supplementary file 2 — Additional file 9: Table S2. Disease names adopted as inclusion criteria. [file 12931_2023_2411_MOESM9_ESM.docx]

Table S2 Disease names adopted as inclusion criteria

| ICD-10 code 1 | disease name 1* | suffix | ICD-10 code 2 | disease name 2** | suffix | emergency admission |
| --- | --- | --- | --- | --- | --- | --- |
| J841 | idiopathic pulmonary fibrosis | AE | - | - | - | - |
| J841 | idiopathic interstitial pneumonia | AE | - | - | - | - |
| J841 | usual interstitial pneumonia | AE | - | - | - | - |
| J841 | pulmonary fibrosis | AE | - | - | - | - |
| J841 | diffuse interstitial pneumonia | AE | - | - | - | - |
| J849 | interstitial pneumonia | AE | - | - | - | - |
| J841 | acute interstitial pneumonia | - | - | - | - | - |
| J841 | diffuse interstitial pneumonia | - | - | - | - | - |
| J841 | diffuse alveolar damage | - | - | - | - | - |
| J80 | acute respiratory distress syndrome | - | - | - | - | - |
| J984 | acute lung injury | - | - | - | - | - |
| J702 | acute drug-induced interstitial lung disorders | - | - | - | - | - |
| J704 | drug-induced interstitial pneumonia | - | - | - | - | - |
| J10〜J111 J15〜189 J690 | pneumonia, Influenza, aspiration pneumonia | - | J841 | idiopathic pulmonary fibrosis | AE | - |
|  |  | - | J841 | idiopathic interstitial pneumonia | AE | - |
|  |  | - | J841 | usual interstitial pneumonia | AE | - |
|  |  | - | J841 | pulmonary fibrosis | AE | - |
|  |  | - | J841 | diffuse interstitial pneumonia | AE | - |
|  |  | - | J841 | interstitial pneumonia | AE | - |
|  |  | - | J841 | idiopathic pulmonary fibrosis | - | - |
|  |  | - | J841 | idiopathic interstitial pneumonia | - | - |
|  |  | - | J841 | usual interstitial pneumonia | - | - |
|  |  | - | J841 | pulmonary fibrosis | - | - |
|  |  | - | J841 | diffuse interstitial pneumonia | - | - |
|  |  | - | J841 | interstitial pneumonia | - | - |
| J841 | idiopathic pulmonary fibrosis | - | - | - | - | ○ |
| J841 | idiopathic interstitial pneumonia | - | - | - | - | ○ |
| J841 | usual interstitial pneumonia | - | - | - | - | ○ |
| J841 | pulmonary fibrosis | - | - | - | - | ○ |
| J841 | diffuse interstitial pneumonia | - | - | - | - | ○ |
| J849 | interstitial pneumonia | - | - | - | - | ○ |

AE, acute exacerbation; DPC, diagnosis procedure combination; ICD-10, the International Statistical Classification of Diseases and Related Health Problems, Tenth Revision

*Disease name in any categories in the DPC data

**Disease name in co-morbidities present at admission and conditions occurring after admission
