# Supplementary material for: Correction: Early corticosteroid dose tapering in patients with acute exacerbation of idiopathic pulmonary fibrosis
Source: Respir Res. 2023 Apr 10;24:107. doi: 10.1186/s12931-023-02411-8 (PMC10084659; doi:10.1186/s12931-023-02411-8)
Supplement: Supplementary file 3 — Additional file 10: Table S3. Disease names adopted as exclusion criteria. [file 12931_2023_2411_MOESM10_ESM.docx]

Table S3 Disease names adopted as exclusion criteria

| ICD-10 code | disease name* |
| --- | --- |
| C00～C96 | malignant neoplasms** |
| D860 | sarcoidosis of lung |
| J60 | coal worker's pneumoconiosis |
| J61 | asbestosis |
| J64 | pneumoconiosis |
| J67 | hypersensitivity pneumonitis due to organic dust |
| J990・M0510 | rheumatoid arthritis-associated interstitial lung disease |
| J991・M351 | collagen vascular disease-associated interstitial lung disease |
| J991・M330 | juvenile dermatomyositis-associated interstitial lung disease |
| J991・M321 | systemic lupus erythematosus-associated interstitial lung disease |
| J991・M332 | polymyositis-associated interstitial lung disease |
| J991・M331 | dermatomyositis-associated interstitial lung disease |
| J991・M313 | lung involvement in granulomatosis with polyangiitis |
| M348 | lung involvement in systemic sclerosis |

* Disease name in any category in the DPC data

**not exclude if they have attached suffix of “post-operation” or “post-radiotherapy”.
